# Supplementary material for: Laparoscopic assisted percutaneous herniorrhapy in dogs using PIRS technique
Source: PLoS One. 2020 Jul 10;15(7):e0235899. doi: 10.1371/journal.pone.0235899 (PMC7351152; doi:10.1371/journal.pone.0235899)
Supplement: Attachment — Submitted filename: New Microsoft Word Document.docx [file pone.0235899.s001.docx]

Dear Editor,

Thank you very much for selecting me fo eviewing the article PONE-D-19-32639.

Dear auther,

Based on reading of the presented study, the article structure is good and the results of origininal research in the veterinary practice and not puplished. The study idea or hypothesis not original as it was introduced in pedeatric surgery for the same point even with the similar topic:

# “Percutaneous internal ring suturing: a simple minimally invasive technique for inguinal hernia repair in children”

- I sugesst to modify your reseach topic.
- I sugesst that the Experiments, statistics, needs more detail using specific statstical programs and tests.
- Did 3 months follow up are enough for juding?
- Is there more clearer ultrasound picture?
- In line 187 & 188, is the ultrasound description of the inguinal hernia “disruption in the continuity of the abdominal wall”. Please make the description revealant with pictures comments.
- The Conclusions are presented in an appropriate fashion and are supported by the recorded data. The article is presented in an intelligible fashion and is written in standard English. The research meets all applicable standards for the ethics of experimentation and research integrit.The article adheres to appropriate reporting guidelines and community standards for data availability.
