# Supplementary material for: Laparoscopic assisted percutaneous herniorrhapy in dogs using PIRS technique
Source: PLoS One. 2020 Jul 10;15(7):e0235899. doi: 10.1371/journal.pone.0235899 (PMC7351152; doi:10.1371/journal.pone.0235899)
Supplement: Attachment — Submitted filename: Comments to the author.docx [file pone.0235899.s002.docx]

Dear authors this topic is very interesting, and the study is well done but unfortunately some major issues must be revised, and some sentences should be edited to meet the journal’s requirements. please find attached the comments.

**Comment 1) I would suggest more attention in the English correction of some words. The authors must check their manuscripts by an English language native speaker before their submission.**

**Some sentences are unclear or complicated. For example:**

Line 40-42, line 55, line 58-60, line 68-72, line 75-76, line 88-89, line 111, line 182-185, line 200-202, line 205-206, line 208-211, line 218-223, line 230-235, line 265-266

**Some sentences need to be revised:**

Line 34. In the literature there are no studies describing … Please reverse it as: In the literature, no studies are describing …

Line 42. Please add a comma after the word inserted

Line 44. It is recommended that the numbers below 10 should be spelled out please revise the sentence in 9 dogs as: in nine dogs.

Line 52. The noun congenital inguinal hernia is missing a determiner before it. Please revise it or change the sentence. For example: In dogs, the congenital inguinal hernia is more common … or Congenital inguinal hernias are more common (Plural)....

Line 53. The noun Acquired inguinal hernia is missing a determiner before it. Please revise it as: An acquired inguinal hernia is more common... or Acquired inguinal hernias are more common ...

Line 58. Please revise the sentence One-sided inguinal hernia is… as: unilateral inguinal hernias are... or the unilateral inguinal hernia is…

Line 62. Please revise the sentence repair of bilateral inguinal hernia as: repair of bilateral inguinal hernias or repair of the bilateral inguinal hernia (The noun is missing a determiner before it)

Line 64. The noun dissection is missing a determiner before it, please change it as: based on the dissection of …

Line 74 and 78. The noun inguinal hernia is missing a determiner before it, please change it as: inguinal hernias or the inguinal hernia

Line 82. Please add an article before the word author’s knowledge: according to the author's knowledge…

Line 94. The noun inguinal hernia is missing a determiner before it, please change it as: inguinal hernias or the inguinal hernia.

Line 99. The first letter of a sentence should be in upper case if multiple sentences are part of the same parentheses. (Resolution no. …)

Line 111. The sentence is correct, but the wording is not optimal please edit this sentence

Line 133. Please revise the word supine position with dorsal recumbency

Line 135. Please add a dot after the word size

Line 137. Please remove the word cavity or revise it as: outside of the abdominal cavity

Line 143. Please add a comma after the word needle

Line 152. Please add a comma after the word loop

Line 153. Please add a comma after the word way.

Line 156. The word umbilicus is missing a determiner, please add.

Line 160. The noun frequency is missing a determiner before it, please add.

Line 162-163. Please revise the sentence the animal was placed on its back in a

positioning trough, as: the animal was placed on the dorsal recumbency.

Line 163. Replace machine with clipper

Line 173. Please add an article before the word optical: where an optical …

Line 182. Please add an article before the word first: by the first intention …

Line 187. Please add an article before the word disruption

Line 189. Please add an article before the word dislocation

Line 198. Please revise the word trocar to the plural form

Line 208. Please add an article before the word introduction. For example: … requires an introduction of …

Line 215. The word doctor is correct, but it would be much better if you replace it with veterinarians or surgeons

Line 220. Please add an article before the word bilateral: a bilateral …

Line 224. Please add an article before the word primary: if a primary

Line 225. Please add an article before the word classical (two times)

Line 226. Please revise the unilateral hernia to the plural form: unilateral hernias …

Line 226. Please delete the word in order. The phrase in order to may be wordy. Consider changing the wording with to.

Line 234. Please revise the word hernia to the pleural form: hernias

Line 236. Please delete the word in order

Line 238. Please revise the sentence presence of inguinal hernia to presentation of an inguinal hernia (The word presence does not seem to fit in this context. Consider replacing it with presentation)

Line 238. Remove the word a please (The indefinite article, a, may be redundant when used with the uncountable noun prevention in your sentence)

Line 241. Please revise the word hernia to the pleural form: hernias

Line 242. Please add an article before the word inguinal: an inguinal

Line 245. Nine should be spelled out

Line 268. Please add a comma after the word trauma

Line 269. Please add a comma after the word functioning

Line. 273. Please add a comma after the word prick

**Comment 2) The number of cases included is low and this can create a statistical problem. Please discuss it.**

**Comment 3) The statistical analysis is missing in this study. For pre-review paper, there must be a statistical analysis. In case there is not so much data or the number of replications is low, the power statistic of the trial should be explained.**

**Comment 4) The clinical and ultrasound examination were done before, immediately after and three months after surgery. Could you please discuss the reason that you choose three months after surgery as the third examination time, and why no long-term follow-up was done!**

**Comment 5) Line 58-60: The contents of the hernial sac may include, among others: omentum, fat, uterus, small intestine, colon, bladder and spleen. Is there any article that reports spleen as a hernial sac content in dogs? If yes, please write the references otherwise revise it; however, the sentence is unclear and should be revised.**

**Comment 6) Please talk about the study limitations.**

**Comment 7) Please discuss the complication rate of the other open surgical and laparoscopic methods in small animals and compare these results with your results and assess the outcomes. Is there any significant difference between these methods? (you reported the results but did not compare it with the control group (for example open surgery vs. laparoscopic method) or results reported by other studies)**

**Comment 8) The discussion is, unfortunately, week and without a strong argument.**

**Comment 9) Line 206-207: The reference number 17 is not explaining the written part.**

**Comment 10) The authors did not talk about the advantages and disadvantages of the open and laparoscopic methods.**

**Comment 11) Line 223-224: The written part is not explaining the mentioned reference correctly (Ref. no. 19).**

Best regards
