# Supplementary material for: Laparoscopic assisted percutaneous herniorrhapy in dogs using PIRS technique
Source: PLoS One. 2020 Jul 10;15(7):e0235899. doi: 10.1371/journal.pone.0235899 (PMC7351152; doi:10.1371/journal.pone.0235899)
Supplement: Attachment — Submitted filename: Response to Reviewers.docx [file pone.0235899.s003.docx]

Dear PLOS ONE Editors, Dear Revivers.

At the beginning I would like to thank you very much for a thorough review of our article under the original title „Percutaneous Internal Ring Suturing - minimally invasive technique for inguinal hernia repair in dogs.” All authors are grateful for the reviewers' recognition of the topic of the article as interesting and useful in minimally invasive surgery in animals.

As requested by the editors, I am sending the revised version of the manuscript. I hope that the improved article will be accepted for publication in PLOS ONE.

Kind regards,

Przemysław Prządka

**Below are the authors 'responses to the reviewers' comments**

Reviewer #1: I read carefully this case series study it is interesting method with laparoscopic assisted herniorrhaphy in dog I think it could be useful and interesting for small animal practitioner and clinicians so I recommend strongly for publication.
I recommend to change the title as "laparoscopic assisted inguinal herniorrhaphy through the percutaneous insertion of injection needle" .

The title of the work has been changed

Reviewer #2: Dear auther,
Based on reading of the presented study, the article structure is good and the results of origininal research in the veterinary practice and not puplished. The study idea or hypothesis not original as it was introduced in pedeatric surgery for the same point even with the similar topic:
“Percutaneous internal ring suturing: a simple minimally invasive technique for inguinal hernia repair in children”

- I sugesst to modify your reseach topic. - The title of the work has been changed
- I sugesst that the Experiments, statistics, needs more detail using specific statstical programs and tests. - Based on the data presented in the article according to statisticians, it is difficult to distribute statistics even with use specific statstical programs and tests.
- Did 3 months follow up are enough for juding? - According to the authors and available literature, sufficient for early evaluation (reference in the discussion), we additionally expanded the observations in the article to 6 months - information from the owners.
- Is there more clearer ultrasound picture? - The authors improved the quality of the ultrasound picture.
- In line 187 & 188, is the ultrasound description of the inguinal hernia “disruption in the continuity of the abdominal wall”. Please make the description revealant with pictures comments. - Done
- The Conclusions are presented in an appropriate fashion and are supported by the recorded data. The article is presented in an intelligible fashion and is written in standard English. The research meets all applicable standards for the ethics of experimentation and research integrit.The article adheres to appropriate reporting guidelines and community standards for data availability

Reviewer #3: Dear authors this topic is very interesting, and the study is well done but unfortunately some major issues must be revised, and some sentences should be edited to meet the journal’s requirements. please find attached the comments.

Comment 1) I would suggest more attention in the English correction of some words. The authors must check their manuscripts by an English language native speaker before their submission. - The article has been improved in terms of language. All reviewer's suggestions have been included in the new version of the manuscript.
Some sentences are unclear or complicated. For example:
Line 40-42, line 55, line 58-60, line 68-72, line 75-76, line 88-89, line 111, line 182-185, line 200-202, line 205-206, line 208-211, line 218-223, line 230-235, line 265-266

Some sentences need to be revised:
Line 34. In the literature there are no studies describing … Please reverse it as: In the literature, no studies are describing …
Line 42. Please add a comma after the word inserted
Line 44. It is recommended that the numbers below 10 should be spelled out please revise the sentence in 9 dogs as: in nine dogs.
Line 52. The noun congenital inguinal hernia is missing a determiner before it. Please revise it or change the sentence. For example: In dogs, the congenital inguinal hernia is more common … or Congenital inguinal hernias are more common (Plural)....
Line 53. The noun Acquired inguinal hernia is missing a determiner before it. Please revise it as: An acquired inguinal hernia is more common... or Acquired inguinal hernias are more common ...
Line 58. Please revise the sentence One-sided inguinal hernia is… as: unilateral inguinal hernias are... or the unilateral inguinal hernia is…
Line 62. Please revise the sentence repair of bilateral inguinal hernia as: repair of bilateral inguinal hernias or repair of the bilateral inguinal hernia (The noun is missing a determiner before it)
Line 64. The noun dissection is missing a determiner before it, please change it as: based on the dissection of …
Line 74 and 78. The noun inguinal hernia is missing a determiner before it, please change it as: inguinal hernias or the inguinal hernia
Line 82. Please add an article before the word author’s knowledge: according to the author's knowledge…
Line 94. The noun inguinal hernia is missing a determiner before it, please change it as: inguinal hernias or the inguinal hernia.
Line 99. The first letter of a sentence should be in upper case if multiple sentences are part of the same parentheses. (Resolution no. …)
Line 111. The sentence is correct, but the wording is not optimal please edit this sentence
Line 133. Please revise the word supine position with dorsal recumbency
Line 135. Please add a dot after the word size
Line 137. Please remove the word cavity or revise it as: outside of the abdominal cavity
Line 143. Please add a comma after the word needle
Line 152. Please add a comma after the word loop
Line 153. Please add a comma after the word way.
Line 156. The word umbilicus is missing a determiner, please add.
Line 160. The noun frequency is missing a determiner before it, please add.
Line 162-163. Please revise the sentence the animal was placed on its back in a
positioning trough, as: the animal was placed on the dorsal recumbency.
Line 163. Replace machine with clipper
Line 173. Please add an article before the word optical: where an optical …
Line 182. Please add an article before the word first: by the first intention …
Line 187. Please add an article before the word disruption
Line 189. Please add an article before the word dislocation
Line 198. Please revise the word trocar to the plural form
Line 208. Please add an article before the word introduction. For example: … requires an introduction of …
Line 215. The word doctor is correct, but it would be much better if you replace it with veterinarians or surgeons
Line 220. Please add an article before the word bilateral: a bilateral …
Line 224. Please add an article before the word primary: if a primary
Line 225. Please add an article before the word classical (two times)
Line 226. Please revise the unilateral hernia to the plural form: unilateral hernias …
Line 226. Please delete the word in order. The phrase in order to may be wordy. Consider changing the wording with to.
Line 234. Please revise the word hernia to the pleural form: hernias
Line 236. Please delete the word in order
Line 238. Please revise the sentence presence of inguinal hernia to presentation of an inguinal hernia (The word presence does not seem to fit in this context. Consider replacing it with presentation)
Line 238. Remove the word a please (The indefinite article, a, may be redundant when used with the uncountable noun prevention in your sentence)
Line 241. Please revise the word hernia to the pleural form: hernias
Line 242. Please add an article before the word inguinal: an inguinal
Line 245. Nine should be spelled out
Line 268. Please add a comma after the word trauma
Line 269. Please add a comma after the word functioning
Line. 273. Please add a comma after the word prick

Comment 2) The number of cases included is low and this can create a statistical problem. Please discuss it. - Made in the discussion.

Comment 3) The statistical analysis is missing in this study. For pre-review paper, there must be a statistical analysis. In case there is not so much data or the number of replications is low, the power statistic of the trial should be explained. - Based on the data presented in the article according to statisticians, it is difficult to distribute statistics even with use specific statstical programs and tests.

Comment 4) The clinical and ultrasound examination were done before, immediately after and three months after surgery. Could you please discuss the reason that you choose three months after surgery as the third examination time, and why no long-term follow-up was done! - According to the authors and available literature, sufficient for early evaluation (reference in the discussion), we additionally expanded the observations in the article to 6 months - information from the owners.

Comment 5) Line 58-60: The contents of the hernial sac may include, among others: omentum, fat, uterus, small intestine, colon, bladder and spleen. Is there any article that reports spleen as an inguinal hernia content in dogs? If yes, please write the references otherwise revise it; however, the sentence is unclear and should be revised. - Literature has been added.

Comment 6) Please talk about the study limitations. - Made in the discussion.

Comment 7) Please discuss the complication rate of the other open surgical and laparoscopic methods in small animals and compare these results with your results and assess the outcomes. Is there any significant difference between these methods? (you reported the results but did not compare it with the control group (for example open surgery vs. laparoscopic method) or results reported by other studies) - Made in the discussion.

Comment 8) The discussion is, unfortunately, week and without a strong argument. - The discussion has been expanded and improved.

Comment 9) Line 206-207: The reference number 17 is not explaining the written part. - This part of the text and literature has been removed from the manuscript.

Comment 10) The authors did not talk about the advantages and disadvantages of the open and laparoscopic methods. - Added to the manuscript.

Comment 11) Line 223-224: The written part is not explaining the mentioned reference correctly (Ref. no. 19). - Corrected in the manuscript.


Best regards

Reviewer #4: The manuscript entitled “Percutaneous Internal Ring Suturing – minimally invasive technique for inguinal hernia repair in dogs” is a first description in dogs (n = 9) of an already described surgical technique for inguinal herniorrhaphy in humans (Percutaneous Internal Ring Suturing). The subject is important for companion animal surgery, due to absence of this the theme in the recent literature and the minimal necessity of expertise in endoscopic surgery and also minimal complications observed during the postoperative period. Additionally, the authors described perfectly clear the surgical technique and images are illustrative and have good quality.
However, the discussion about the advantages of the technique is weak, lacking comparison with previous literature about surgical time, postoperative pain and even costs. Also, the conclusions are poorly established in consequence of weak discussion and small number of cases.- The discussion has been expanded and improved.
Although there are no groups to be compared, the means are shown with standard deviation, but no normality tests of the data were mentioned by the authors. - Based on the data presented in the article according to statisticians, it is difficult to distribute statistics even with use specific statstical programs and tests.
The authors didn't expose the limitations of their study and technique, probably the size of the dog and weight should be part of discussion and limitations as well. - Corrected and added in the manuscript.
This is a good quality material and could be submitted as short communication elsewhere or even in Plos One as "Registered Report Protocol" if the authors intend to do future study comparing the different techniques.

Reviewer #5: Abstract in this form is need to be rearranged, please divide it into background, aim, Methods, results and conclusions. - Corrected in the manuscript.
Line 40: do you mean “through” instead of trough.
line 41: the line need to rephrased because it's confusing"including, 18-gauge injection needle with non absorbable 2-0 poly filament"
line 42: please explain that more than one dog was suffering from bilateral inguinal hernia therefore operation done on 9 dogs
abstract stated no conclusions please add it
introduction
line 54: Waters etal 1993 referred to 16 dogs of 22 dogs 73% older than 2 years were female, please rephrase.
line 61-67: please describe other complications of open surgery approach that make laparoscopic approach more safer.
line 69:what reports please insert citation and clarify if these reports "experimental study or clinical cases" and if later applied,please describe what make this research unique.
line 74-77: it need to be rearranged to come before paragraph starting at line 69.
line 78: This part need to be described more extensively as this is the first report of PIRS in dogs and this is very interesting aspect.
line 82- 91: there are a lot of redundancy please rewrite.
line 83:This is the only laparoscopic................etc to tissue injury, please rephrase or add till now.
please add clear aim at the end of introduction part.

Material&Methods - Materials and methods have been improved as suggested by the reviewer.
please add inclusion criteria for this research
line 107-109: belong to results section.
line 111: is confusing , do you mean (table1) or Is there missing word after on?
line 114: what's current blood test? please, specify and omit "there were no contraindications to general anesthesia"
line 122: please add an approximate time of operation including anesthesia and Pneumoperitoneum.
line 131-132: no need to re-emphasize that procedure was done under anesthesia.
line 133: please add in bracket gas that used (co2)
please add in bracket brand of insufflation device.
line 141: please specify in details site of needle insertion.
line 158: the subsection need to be rearranged before anesthesia.
The operative technique is extensively described as expected and it's one of this paper strength.

Results: - The results have been improved as suggested by the reviewer.
line 168: What do you mean with "without necessity to conversion"
line 171: Is this time including induction of Pneumoperitoneum, if so please,add in a bracket.
please rearrange ultrasound findings to be after first paragraph.
line 181: please add approximate percentage of possible complications.
Figures are excellent and captions are descriptive.

Discussion: - The whole discussion has been corrected as suggested by the reviewer
line 210: 12G or 18G
in general you should state your findings and compare them with other reports, and describe why this technique is superior to other technique.
for example : this approach requires one port and other need 2-3 ports, reduce expected trauma, complications......etc.
again there is no conclusions at end of discussion.
you need to state "a take home message", do you use of this procedure instead of common surgical technique.
what are expected limitations of this technique?
is it cost effective compared to traditional route?
what are expected complications compared to traditional technique?
Are there contraindications to this technique other than known contraindications for traditional surgical practice ?
